# Evaluation of Frequency of Encounters With Primary Care Physicians vs Visits to Community Pharmacies Among Medicare Beneficiaries

**DOI:** 10.1001/jamanetworkopen.2020.9132

**Published:** 2020-07-15

**Authors:** Lucas A. Berenbrok, Nico Gabriel, Kim C. Coley, Inmaculada Hernandez

**Affiliations:** 1 Department of Pharmacy and Therapeutics, University of Pittsburgh School of Pharmacy, Pittsburgh, Pennsylvania

## Abstract

**Importance:**

The shift toward value-based care has placed emphasis on preventive care and chronic disease management services delivered by multidisciplinary health care teams. Community pharmacists are particularly well positioned to deliver these services due to their accessibility.

**Objective:**

To compare the number of patient visits to community pharmacies and the number of encounters with primary care physicians among Medicare beneficiaries who actively access health care services.

**Design, Setting, and Participants:**

This cross-sectional study analyzed a 5% random sample of 2016 Medicare beneficiaries from January 1, 2016, to December 31, 2016 (N = 2 794 078). Data were analyzed from October 23, 2019, to December 20, 2019. Medicare Part D beneficiaries who were continuously enrolled and had at least 1 pharmacy claim and 1 encounter with a primary care physician were included in the final analysis (n = 681 456). Those excluded from the study were patients who were not continuously enrolled in Part D until death, those with Part B skilled nursing claims, and those with Part D mail-order pharmacy claims.

**Exposures:**

We conducted analyses for the overall sample and for subgroups defined by demographics, region of residence, and clinical characteristics.

**Main Outcomes and Measures:**

Outcomes included the number of visits to community pharmacies and encounters with primary care physicians. Unique visits to the community pharmacy were defined using a 13-day window between individual prescription drug claims. Kruskal-Wallis tests were used to compare the medians for the 2 outcomes.

**Results:**

A total of 681 456 patients (mean [SD] age, 72.0 [12.5] years; 418 685 [61.4%] women and 262 771 [38.6%] men) were included in the analysis; 82.2% were white, 9.6% were black, 2.4% were Hispanic, and 5.7% were other races/ethnicities. Visits to the community pharmacy outnumbered encounters with primary care physicians (median [interquartile range (IQR)], 13 [9-17] vs 7 [4-14]; *P* < .001). The number of pharmacy visits was significantly larger than the number of primary care physician encounters for all subgroups evaluated except for those with acute myocardial infarction (median [IQR], 15 [12-19] vs 14 [7-26]; *P* = .60 using a 13-day window). The difference in the number of pharmacy and primary care physician encounters was larger in rural areas (median [IQR], 14 [10-17] vs 5 [2-11]; *P* < .001) than in metropolitan areas (median [IQR], 13 [8-17] vs 8 [4-14]; *P* < .001). In all 50 states and in all but 9 counties, the number of community pharmacy visits was larger than the number of encounters with primary care physicians.

**Conclusions and Relevance:**

This cross-sectional study suggests that community pharmacists are accessible health care professionals with frequent opportunities to interact with community-dwelling patients. Primary care physicians should work collaboratively with community pharmacists, who can assist in the delivery of preventive care and chronic disease management.

## Key Points

**Question:**

How often do Medicare beneficiaries who actively access health care services visit community pharmacies compared with primary care physicians?

**Findings:**

Among the 681 456 active Medicare beneficiaries included in this nationwide cross-sectional study, the median number of visits to community pharmacies was significantly higher than encounters with primary care physicians (13 vs 7).

**Meaning:**

These findings suggest that community pharmacists are accessible health care professionals with frequent opportunities to deliver preventive care and chronic disease management services in collaboration with primary care physicians.

## Teaser

This cross-sectional study uses 2016 Medicare claims data to assess the frequency of patient encounters with community pharmacists compared with primary care physicians for preventive care and chronic disease management.

## Introduction

The shift toward value-based care has placed emphasis on preventive care and chronic disease management services delivered by multidisciplinary health care teams. However, some patients continue to have difficulty accessing affordable quality care. Pharmacists are accessible and trusted members of the health care team and routinely encounter patients at their community pharmacies. Patient-pharmacist encounters have traditionally focused on the provision of medications. More recently, community pharmacists have transformed and optimized their roles from product-centered services (ie, medication dispensing and sale of over-the-counter medications) to patient-centered services (ie, medication therapy management). The goal of medication therapy management is to optimize medication use, reduce the risk of adverse events, and improve medication adherence.^1^

Beyond the provision of medication therapy management services, pharmacists actively contribute to affordable quality care by offering preventive care services, such as administering vaccinations and identifying patients at high risk for certain diseases.^2^ Pharmacists have also shown positive effects on patient and medication outcomes when contributing to the management of chronic diseases, including diabetes (type 1 and type 2), hypertension, hyperlipidemia, asthma, and depression.^3,4,5,6,7^

To understand the potential for pharmacist-delivered preventive services and chronic care management, it is important to quantify how many times patients are likely to encounter community pharmacists and how this frequency compares with the number of patient encounters with primary care physicians. Previously, Tsuyuki et al^8^ performed a nonsystematic review and found that patients encountered pharmacists between 1.5 and 10 times more frequently than they encountered primary care physicians. However, this nonsystematic review included only 1 study from the United States, and it was not peer reviewed.^8^ To our knowledge, there is no peer-reviewed literature to date comparing the frequency of patient visits to community pharmacies with the number of patient encounters with primary care physicians in the United States. To address this evidence gap, we used 2016 data from a nationally representative sample of Medicare beneficiaries who actively access health care services.

## Methods

### Data Source and Study Sample

**Figure 1.**
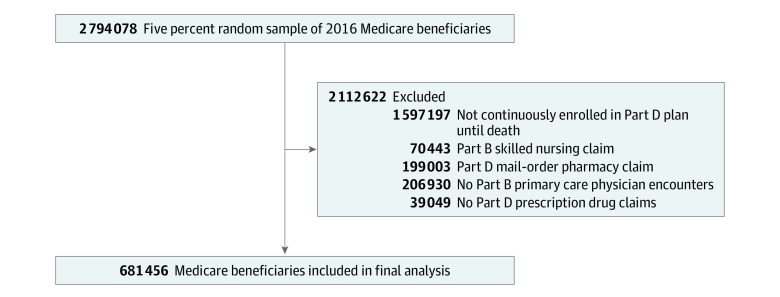
Flow Diagram

For this cross-sectional study, we obtained 2016 claims data from a 5% random sample of Medicare Part D beneficiaries from the Centers for Medicare and Medicaid Services from January 1, 2016, to December 31, 2016 (N = 2 794 078). Figure 1 provides an overview of the sample selection. We selected beneficiaries continuously enrolled in Medicare Part D in 2016 or until death. Beneficiaries with Medicare Part B skilled nursing claims or at least 1 Part D prescription drug claim from a mail-order pharmacy were excluded, because these patients have markedly fewer opportunities to visit a community pharmacy. For the sample to be representative of patients who actively access health care professionals, we only included beneficiaries who had at least 1 Part D prescription drug claim and at least 1 encounter with a primary care physician in 2016. Encounters with primary care physicians were identified using health care provider claims. The final sample included 681 456 Medicare beneficiaries. This study was approved by the University of Pittsburgh institutional review board as exempt from obtaining patient consent because deidentified data were used in the analyses. This study followed the Strengthening the Reporting of Observational Studies in Epidemiology (STROBE) reporting guideline.

### Outcomes

Outcomes included the number of encounters with primary care physicians and with community pharmacies and were expressed per person-year. Primary care physician encounters included encounters with physicians whose specialty was identified as family practice, general practice, geriatric medicine, internal medicine, or preventive medicine from Part B health care provider claims. Visits to the community pharmacy were estimated using Part D pharmacy claims. We defined unique visits to the community pharmacy using a 13-day window between individual prescription drug claims. In other words, prescription drug claims less than 13 days apart were considered the same pharmacy visit. A 13-day window was used because most pharmacy benefit managers require pharmacies to reverse claims for medications not picked up by the patient within 14 days of initial claim submission. This means that individual prescriptions with paid claims separated by 14 days or more would require 2 unique visits to the pharmacy. A 13-day window also allowed us to group multiple prescription drug claims synchronized around the same pickup date into 1 pharmacy visit. In sensitivity analyses, we used a 10-day window because 1 national community pharmacy chain reverses claims for medications not picked up by the patient within 10 days of initial claim submission.

### Independent Variables

Independent variables of interest included demographics, region of residence, and clinical characteristics. Demographic characteristics included age, sex, and race/ethnicity. Region of residence variables included degree of urbanization, an indicator variable for medically underserved area designation, state, and county and were all defined using the Federal Information Processing System code for each beneficiary. We used the 2013 Rural-Urban Continuum Codes from the US Department of Agriculture Economic Research Service to categorize Federal Information Processing System codes into 3 levels of urbanization, including metropolitan areas (codes 1-3), nonmetropolitan urban areas (codes 4-7), and nonmetropolitan rural areas (codes 8-9).^9^ To identify medically underserved areas, we used data from the Health Resources & Services Administration.^10^ Clinical characteristics included a history of acute myocardial infarction, asthma, atrial fibrillation, chronic kidney disease, chronic obstructive pulmonary disease, depression, diabetes (type 1 and type 2), heart failure, hyperlipidemia, hypertension, osteoporosis, rheumatoid arthritis or osteoarthritis, and stroke or transient ischemic attack. Clinical characteristics were defined using the Centers for Medicare and Medicaid Services Chronic Condition Data Warehouse definitions.^11^

### Statistical Analysis

We compared the median number and interquartile ranges (IQRs) of encounters with primary care physicians and visits to the community pharmacy using Kruskal-Wallis tests. We conducted analyses for the overall sample and for subgroups defined by the independent variables listed previously. To explore whether there was geographic variation in the frequency of encounters with primary care physicians and visits to community pharmacies, we reported the differences in the median number of encounters by state and by county. Data were analyzed from October 23, 2019, to December 20, 2019. Analyses were conducted using SAS, version 9.4 (SAS Institute Inc) and R, version 3.6.1 (R Project for Statistical Computing). Two-sided *P* values were used. Statistical significance was set at *P* < .05.

## Results

### Patient Characteristics

Table 1 shows baseline demographic and clinical characteristics of the study sample. A total of 681 456 patients (mean [SD] age, 72.0 [12.5] years; 418 685 women [61.4%] and 262 771 men [38.6%]) were included in the analysis; 82.2% were white, 9.6% were black, 2.4% were Hispanic, and 5.7% were other races/ethnicities. Of the total number of patients, 289 482 patients (42.5%) were 75 years or older, 271 546 (39.9%) were aged 65 to 74 years, and 120 428 (17.7%) were younger than 65 years. Less than one-quarter of the study beneficiaries (160 591 [23.6%]) lived in medically underserved areas.

**Table 1.** Characteristics of Active Medicare Beneficiaries Included in Analysis

Variable	No. (%)
Age, y	
<65	120 428 (17.7)
65-74	271 546 (39.9)
≥75	289 482 (42.5)
Sex	
Male	262 771 (38.6)
Female	418 685 (61.4)
Race/ethnicity	
White	560 416 (82.2)
Black	65 469 (9.6)
Hispanic	16 567 (2.4)
Other	39 004 (5.7)
Region of residence	
By degree of urbanization^a^^,^^b^	
Metropolitan area	529 414 (77.7)
Nonmetropolitan area	
Urban	134 692 (19.8)
Rural	16 537 (2.4)
By access to health care^c^	
Medically underserved area	160 591 (23.6)
Nonmedically underserved area	520 865 (76.4)
By region of residence^b^	
Northeast	128 997 (18.9)
Midwest	153 577 (22.5)
South	282 809 (41.5)
West	114 765 (16.8)

^a^ We used Rural-Urban Continuum Codes from the US Department of Agriculture Economic Research Service to categorize metropolitan areas (codes 1-3), nonmetropolitan urban areas (codes 4-7), and nonmetropolitan rural areas (codes 8-9).^9^

^b^ Does not sum to group totals due to missing data.

^c^ We used data from the Health Resources & Services Administration to identify medically underserved areas.^10^

### Primary Results

Overall visits to the community pharmacy significantly outnumbered encounters with primary care physicians (median [IQR], 13 [9-17] vs 7 [4-14]; *P* < .001) using the conservative 13-day prescription drug claim window (Table 2). The difference between community pharmacy visits and primary care physician encounters increased (median [IQR], 14 [9-19] vs 7 [4-14]; *P* < .001) when the less conservative 10-day prescription drug claim window was applied.

**Table 2.** Number of Primary Care Physician Encounters and Pharmacy Visits per Person-Year for the Overall Sample and by Subgroups

Variable	No. of encounters/visits per person-year, median (IQR)			*P* value for comparison between primary care physician encounters and pharmacy visits	
	Primary care physician encounters	Pharmacy visits			
		Defined using 13-d window	Defined using 10-d window	Defined using 13-d window	Defined using 10-d window
Overall	7 (4-14)	13 (9-17)	14 (9-19)	<.001	<.001
Subgroup analyses demographics					
Age, y					
<65	7 (3-13)	15 (10-18)	16 (11-21)	<.001	<.001
65-74	7 (3-13)	12 (8-16)	13 (8-18)		
≥75	8 (4-15)	13 (9-17)	14 (10-19)		
Sex					
Male	7 (3-13)	13 (8-17)	14 (9-19)	<.001	<.001
Female	8 (4-14)	13 (9-17)	14 (9-19)		
Race					
White	7 (4-14)	13 (9-17)	14 (9-19)	<.001	<.001
Black	7 (3-14)	13 (9-17)	15 (10-20)		
Hispanic	8 (4-15)	13 (9-17)	14 (9-19)		
Other	7 (4-14)	12 (7-15)	13 (8-17)		
Region of residence					
By degree of urbanization^a^					
Metropolitan area	8 (4-14)	13 (8-17)	14 (9-19)	<.001	<.001
Nonmetropolitan area					
Urban	6 (3-12)	14 (10-17)	15 (10-20)	<.001	<.001
Rural	5 (2-11)	14 (10-17)	15 (10-20)		
By access to health care^b^					
Medically underserved area	8 (4-14)	13 (8-17)	14 (9-19)	<.001	<.001
Nonmedically underserved area	7 (3-13)	14 (9-17)	15 (10-20)		
Clinical characteristics^c^					
Acute myocardial infarction	14 (7-26)	15 (12-19)	17 (12-21)	.60	<.001
Asthma	12 (6-21)	16 (12-19)	18 (13-22)	<.001	
Atrial fibrillation	13 (7-23)	16 (12-19)	18 (13-22)		
Chronic kidney disease	11 (5-20)	15 (12-19)	17 (12-22)		
COPD	12 (6-21)	16 (12-19)	18 (13-22)		
Depression	10 (5-18)	16 (12-19)	18 (13-22)		
Diabetes	10 (5-17)	15 (11-18)	17 (12-21)		
Heart failure	10 (5-19)	15 (11-18)	17 (12-21)		
Hyperlipidemia	9 (5-16)	14 (10-17)	15 (11-20)		
Hypertension	9 (5-16)	14 (10-18)	15 (11-20)		
Osteoporosis	11 (6-18)	14 (10-18)	15 (10-20)		
Rheumatoid arthritis/osteoarthritis	10 (5-18)	14 (11-18)	16 (11-21)		
Stroke or transient ischemic attack	13 (7-23)	15 (11-18)	17 (12-21)		

Abbreviations: COPD, chronic obstructive pulmonary disease; IQR, interquartile range.

^a^ We used Rural-Urban Continuum Codes from the US Department of Agriculture Economic Research Service to categorize metropolitan areas (codes 1-3), nonmetropolitan urban areas (codes 4-7), and nonmetropolitan rural areas (codes 8-9).^9^

^b^ We used data from the Health Resources & Services Administration to identify medically underserved areas.^10^

^c^ We used the Centers for Medicare and Medicaid Services Chronic Condition Data Warehouse definitions of priority conditions.^11^

### Results of Subgroup Analysis

Using 13-day windows to define pharmacy visits, the difference between community pharmacy visits and primary care physician encounters was greater for beneficiaries living in rural areas (median [IQR], 14 [10-17] vs 5 [2-11]; *P* < .001) than for beneficiaries living in metropolitan areas (median [IQR], 13 [8-17] vs 8 [4-14]; *P* < .001) (Table 2). The number of community pharmacy visits was statistically larger than the number of primary care physician encounters for all clinical characteristic subgroups evaluated except for beneficiaries with acute myocardial infarction (median [IQR], 15 [12-19] vs 14 [7-26]; *P* = .60). When the less conservative 10-day window was applied, community pharmacy visits were also significantly greater than primary care physician encounters for beneficiaries with a history of acute myocardial infarction (median [IQR], 17 [12-21] vs 14 [7-26]; *P* < .001). Using 13-day windows to define pharmacy visits, differences between community pharmacy visits and primary care physician encounters were greatest for beneficiaries with depression (median [IQR], 16 [12-19] vs 10 [5-18]; *P* < .001). Beneficiaries with chronic disease states related to metabolic syndrome, including diabetes (type 1 and type 2), hyperlipidemia, and hypertension, visited the pharmacy 5 occasions more than the primary care physician: diabetes (median [IQR], 15 [11-18] vs 10 [5-17]; *P* < .001), heart failure (median [IQR], 15 [11-18] vs 10 [5-19]; *P* < .001), hyperlipidemia (median [IQR], 14 [10-17] vs 9 [5-16]; *P* < .001), and hypertension (median [IQR], 14 [10-18] vs 9 [5-16]; *P* < .001).

**Figure 2.**
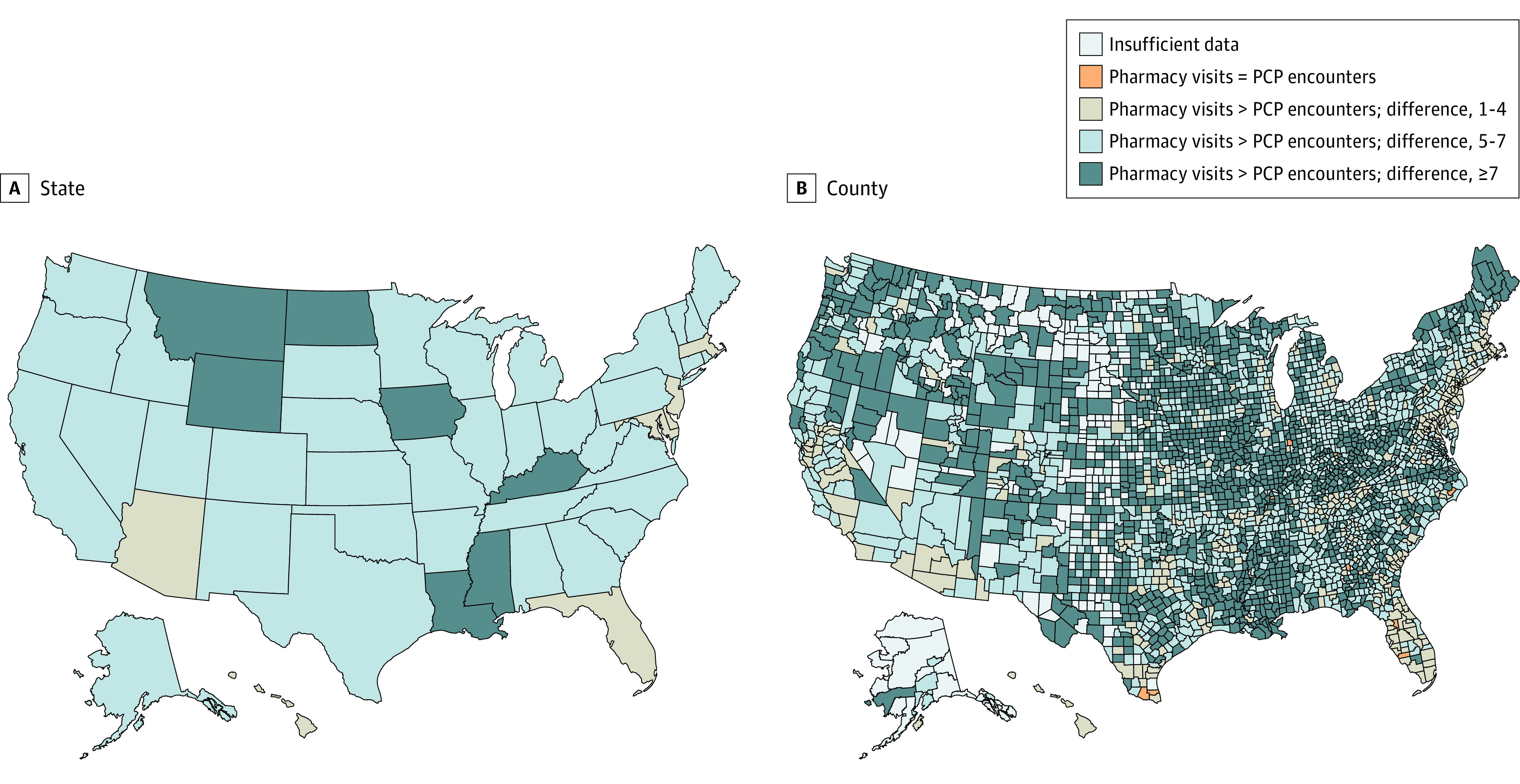
Difference in the Median Number of Encounters With Primary Care Physicians (PCPs) and Visits to Community Pharmacies This figure represents the difference between the median number of visits to the community pharmacy and encounters with primary care physicians by state (A) and by county (B). Pharmacy visits were defined using a 13-day window between claims, as explained in the Methods section. Insufficient data denotes that there were less than 11 beneficiaries in each county, which is the minimum cell size requirement for reporting from the Centers for Medicare and Medicaid Services. Only 9 counties had primary care physician encounters that equaled or outnumbered pharmacy visits. These counties are in Florida, Georgia, Indiana, Kentucky, North Carolina, and Texas.

In all 50 states, the number of community pharmacy visits was larger than the number of encounters with primary care physicians (Figure 2A). The difference between community pharmacy visits and primary care physician encounters was largest in Iowa (13 vs 5; *P* < .001), Kentucky (14 vs 7; *P* < .001), Louisiana (15 vs 6; *P* < .001), Mississippi (15 vs 6; *P* < .001), Montana (12 vs 4; *P* < .001), North Dakota (12 vs 4; *P* < .001), and Wyoming (12 vs 4; *P* < .001) and lowest in Arizona (11 vs 8; *P* < .001), Delaware (12 vs 8; *P* < .001), Florida (13 vs 9; *P* < .001), Hawaii (11 vs 8; *P* < .001), Maryland (12 vs 8; *P* < .001), Massachusetts (13 vs 9; *P* < .001), and New Jersey (12 vs 9; *P* < .001). The number of community pharmacy visits was larger than the number of encounters with primary care physicians in all but 9 US counties where primary care physician encounters equaled or outnumbered pharmacy visits, including Charlotte County, Florida (median pharmacy visits, 11 vs median primary care physician encounters, 11); Sumter County, Florida (11 vs 12); Marion County, Georgia (10 vs 10); Parke County, Indiana (13 vs 13); Bracken County, Kentucky (14 vs 14); Carlisle County, Kentucky (6 vs 12); Pamlico County, North Carolina (13 vs 14); Hidalgo County, Texas (14 vs 14); and Willacy County, Texas (14 vs 14) (Figure 2B).

## Discussion

In this cross-sectional study using a nationally representative sample of Medicare beneficiaries who actively access health care services, we found that patients visited community pharmacies approximately twice as frequently as they visited primary care physicians. The difference in frequency of visits and encounters was largest in nonmetropolitan rural areas.

Our study is an important contribution to the literature because it is the first, to our knowledge, to quantify and compare frequency of visits with community pharmacies and primary care physicians in a nationally representative sample. Although most patients visit community pharmacies for product-centered services, including prescription medication procurement and self-care with nonprescription medications, the frequency of visits estimated by our analysis suggests that community pharmacists have frequent opportunities to deliver patient-centered services in community-based locations. As value-based health care increasingly places emphasis on preventive care and chronic disease management, the community pharmacist is a highly accessible clinician who can provide many of these services.

The greatest difference between community pharmacy visits and primary care physician encounters was observed in nonmetropolitan rural areas, which underscores the importance of accessible health care professionals in small or isolated communities. As the need for primary care physicians continues to rise across the United States and particularly in rural areas, pharmacists are well placed to contribute to a multidisciplinary primary care team with direct and frequent follow-up. Frequent follow-up is often needed in the context of chronic disease and preventive medicine.

It is also important to note that pharmacists cannot capitalize on accessible and frequent encounters at community pharmacies without further practice change and transformation. The need to recognize pharmacists as providers of billable services, integrate pharmacists in emerging delivery and payment models, and enhance collaborative relationships between pharmacists and other members of the health care team have been well described in the literature.^12^ To further capitalize on the uniqueness on the pharmacist as an accessible health care professional, pharmacy and health care organizations must consider how community pharmacy practice will adapt to transformed pharmacist roles, including changes to business models, workflows, and staffing.

### Limitations

The findings of our study should be interpreted in the context of its limitations. First, Medicare Part B claims data do not expressly identify the patient’s primary care physician. To minimize this limitation, we included all Part B claims billed to primary care specialties. Our attempt to be comprehensive may have overestimated the number of times patients encountered the primary care physician responsible for comprehensive care for the individual.

Second, we may have underestimated the number of visits to the community pharmacy in the absence of point-of-sale data from community pharmacies. To proceed without point-of-sale data confirming physical presence at the community pharmacy, we defined pharmacy visits using days between individual Part D prescription drug claims with conservative 10- and 13-day windows informed by industry standards. It was not possible to differentiate the number of days used to reverse unclaimed prescriptions at each pharmacy location because of the lack of reversed claims in the data set.

Third, cognitive services provided by pharmacists at the community pharmacy were not captured in our analysis. Pharmacists provide billable services outside of medication dispensing that include immunizations and medication therapy management. Nonbillable services are also routinely offered by community pharmacists at no charge. These include patient education and counseling for prescription medications, recommendations for self-care and nonprescription medications, point-of-care testing for acute and chronic illness, and screening and brief intervention for substance use disorders.

Lastly, our data do not capture the nature of each primary care physician encounter and pharmacy visit. Therefore, inferences as to what preventive service and disease state management were addressed at each primary care encounter or pharmacy visit cannot be made. Likewise, it was not possible to differentiate between health care professional–initiated and patient-initiated visits. Although most pharmacy visits are probably patient initiated, health care professional encounters most likely include a mix of visits initiated by both patients and health care professionals as follow-up care.

## Conclusions

The findings of this cross-sectional study suggest that community pharmacists are accessible health care professionals who have frequent opportunities to interact with patients in the community. Given these findings, we believe community pharmacists and primary care physicians should collaborate as multidisciplinary primary care teams to prevent and manage chronic disease.
